# P-1579. Prediction of Prognosis of Visceral and Subcutaneous Fat Areas in Pyelonephritis

**DOI:** 10.1093/ofid/ofae631.1746

**Published:** 2025-01-29

**Authors:** Takatoshi Kitazawa, Shin Nakayama, Yoshitaka Wakabayashi

**Affiliations:** Teikyo University School of Medicine, Itabashi-ku, Tokyo, Japan; Teikyo University School of Medicine, Itabashi-ku, Tokyo, Japan; Teikyo University School of Medicine, Itabashi-ku, Tokyo, Japan

## Abstract

**Background:**

Visceral fat accumulation has been regarded as a poor prognostic factor for coronary artery disease. Moderate overweight and mild obesity are associated with a lower mortality in patients with several infectious diseases on intensive care units, and this phenomenon is referred to as the obesity paradox. Pyelonephritis is one of the common infectious diseases, and although computed tomography (CT) imaging is frequently used in its localization, its prognostic value is limited. In this study, we analyze the relationship between the visceral and subcutaneous fat areas in CT scans and prognosis of patients with pyelonephritis.

**Fig. 1:**
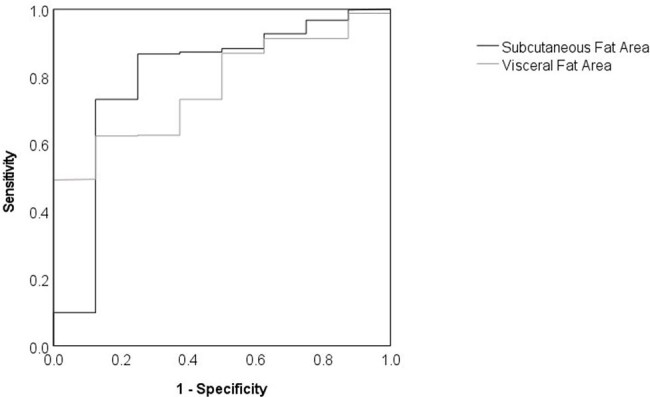
ROC curves using subcutaneous and visceral fat area to discriminating surviving pyelonephritis patients from deceased pyelonephritis patients The black line shows the curve using subcutaneous fat area and the gray line shows the curve using visceral fat area.

**Methods:**

We included hospitalized patients with pyelonephritis who underwent computed tomography of the abdomen. Clinical data were obtained from the medical records. Subcutaneous and visceral fat aeras were analyzed at the umbilical level using the abdominal analysis (2D) function of the 3D image analysis system volume analyzer.

**Results:**

505 patients were included in the study. There were 497 survivors and 8 non-survivors, with both subcutaneous and visceral fat areas being lower in the non-survivors than in the survivors (subcutaneous fat area 49.3 ± 73.9 cm^2^ vs 123.7 ± 85.3 cm^2^, p=0.012; visceral fat area 42.2 ± 29.2 cm^2^ vs 98.4 ± 69.1 cm^2^, p< 0.01, respectively. ROC analysis using subcutaneous and visceral fat areas to discriminate survival and death showed that the areas under the curve of subcutaneous and visceral fat areas were 0.794 (p=0.002) and 0.770 (p< 0.001), respectively (Fig.1).

**Conclusion:**

Patients with poor prognosis of pyelonephritis tended to have lower subcutaneous and visceral fat area at onset than patients with improved prognosis. The results suggest that both subcutaneous and visceral fat area using CT may have prognostic value for pyelonephritis.

**Disclosures:**

**All Authors**: No reported disclosures

